# Long-Term Surveillance and Laparoscopic Management of Zinner Syndrome

**DOI:** 10.1155/2021/6626511

**Published:** 2021-03-09

**Authors:** Niall P. Kelly, Adrian Fuentes-Bonachera, William P. Shields, Ivor M. Cullen, Padraig J. Daly

**Affiliations:** Department of Urology, University Hospital Waterford, Waterford, Ireland

## Abstract

Zinner syndrome was first described in 1914 and represents the triad of unilateral renal agenesis and ipsilateral seminal vesicle cyst and ipsilateral ejaculatory duct obstruction. Seminal vesicle cysts are often asymptomatic but can also present with pain, haematospermia, or other lower urinary tract symptoms. Treatment strategies include observation and surgical excision. We present the laparoscopic management of an enlarged seminal vesicle cyst, consistent with Zinner syndrome, 14 years after the initial diagnosis. A 58-year-old male patient was diagnosed with a left-sided seminal vesicle cyst while undergoing assessment for renal transplant due to progressively worsening renal function in his solitary right kidney. The otherwise asymptomatic cyst enlarged from the time of initial diagnosis in 2004 (11.3 cm × 9.7 cm × 13.1 cm) to nearly double the size in 2018 (12.8 cm × 11.9 cm × 14.2 cm). This cyst size ultimately precluded renal transplant, and the patient was referred for excision. Laparoscopic excision of the cyst was performed, histopathology confirmed seminal vesicle cyst tissue, and there has been no recurrence of the cyst to date. The patient remains active on the renal transplant waitlist. Zinner syndrome is a rare syndrome, with the seminal vesicle cysts being managed by observation or surgical excision. We report the longest documented observation of a seminal vesicle cyst, culminating in a safe and successful laparoscopic excision.

## 1. Introduction

Zinner syndrome was first described in 1914 and represents the triad of unilateral renal agenesis and ipsilateral seminal vesicle cyst and ipsilateral ejaculatory duct obstruction. Often asymptomatic, it can present with haematuria or haematospermia or other lower urinary tract symptoms. The cysts can become markedly enlarged, requiring excision due to symptoms. We present the laparoscopic management of an enlarged seminal vesicle cyst, consistent with Zinner syndrome.

## 2. Case Report

**Figure 1:**
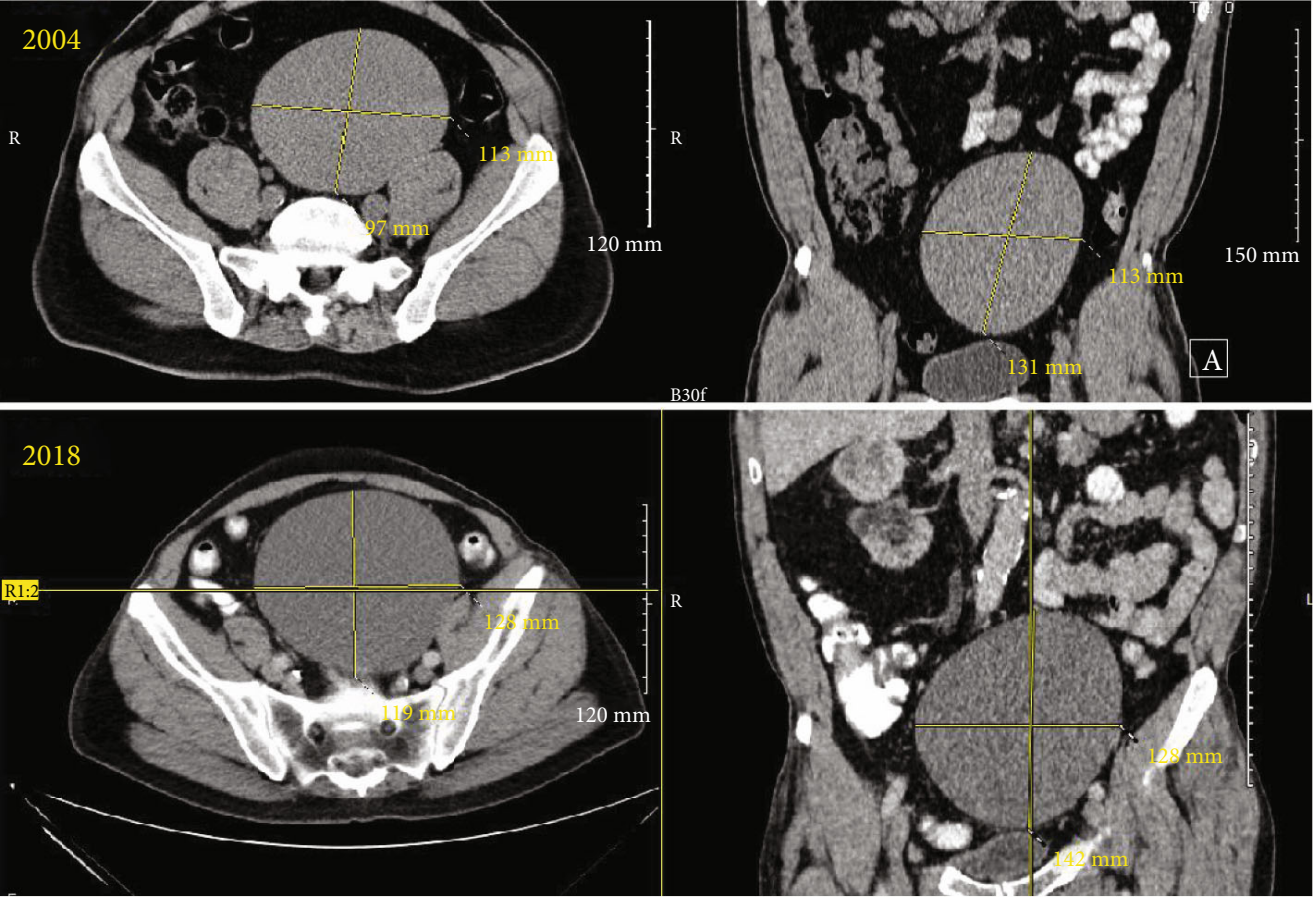
CT, axial, and coronal sections of the seminal vesicle cyst from 2004 and 2018. In 2004, the cyst measured 11.3 cm × 9.7 cm × 13.1 cm (volume approximately 500 cm^3^), while in 2018, it measured 12.8 cm × 11.9 cm × 14.2 cm (volume approximately 900 cm^3^).

A 58-year-old male patient with end-stage renal failure (ESRF) due to assumed reflux nephropathy in a solitary right kidney was undergoing assessment for renal transplantation. His past medical history was significant for non-Hodgkin's lymphoma, treated with chemotherapy (completed in 2014) and hypertension. His baseline serum creatinine at the time of initial referral in 2018 was 522 *μ*g/L. Computer tomography (CT) imaging of the abdomen and pelvis (performed in October 2018) revealed a large cystic lesion arising from the area of the left seminal vesicle in 2018 (12.8 cm × 11.9 cm × 14.2 cm, volume approximately 900 cm^3^) (Figure 1). The patient was referred to our unit for consideration of excision of the cyst as it was deemed too large to allow renal transplantation by the transplant team.

**Figure 2:**
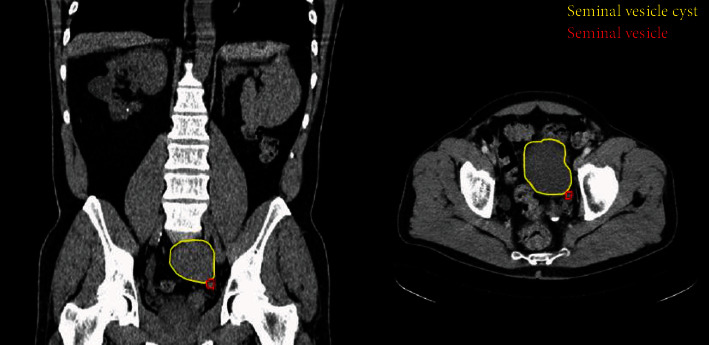
CT, axial, and coronal sections from scan in 2004, of a more inferior aspect of the cyst (outlined in yellow), identifying its relationship to the seminal vesicle (outlined in red).

The seminal vesicle cyst was first diagnosed incidentally following ultrasound examination of the abdomen in 2004 as part of investigations of chronic kidney disease (CKD; serum creatinine in 2004 was 161 *μ*g/L with evidence of proteinuria). This ultrasound identified an absent left kidney and scarring of the right kidney, suggestive of reflux nephropathy, as well as the cyst within the pelvis. The cyst was then assessed with CT, at which time it measured 11.3 cm × 9.7 cm × 13.1 cm (volume approximately 500 cm^3^) within the left side of the pelvis, seen to be originating from the seminal vesicle on that side (Figure 2). This CT also showed some prominence of the right ureter but without a cause for obstruction. Micturating cystourethrogram (MCUG) was not performed during the initial nephrology consultations; thus, reflux could not be absolutely confirmed as the cause of the CKD. As the patient was asymptomatic at the time of initial diagnosis, a decision was taken to defer intervention until only such time that he did become symptomatic. No specific radiological follow-up regimen was followed, although the patient had regular clinical and physical examinations. There was an approximate 80% increase in the volume of the cyst over the 14-year time period, leading to a large lesion occupying the pelvis and precluding safe transplantation of a donor kidney.

The patient underwent laparoscopy with excision of the seminal vesicle cyst in January 2019. A 3-port set-up was used with the patient in a slight Trendelenburg position. The colon was mobilised out of the pelvis, and an impression of the large cyst could be seen in the floor of the peritoneum. The peritoneum was incised and dissected from the cyst. The cyst was incised and drained of approximately 400 mls dark fluid; no organisms grew on the culture of this. Drainage of the cyst allowed for better visualisation of the adherent areas as well as location of the base of the cyst. The specimen was retrieved, and histopathology confirmed that the cyst was of seminal vesicle origin. The patient had an uneventful postoperative stay and was discharged on the first postoperative day. There has been no recurrence of the cyst to date, and he was referred to the nephrological service where he remains active on the renal transplant waitlist.

## 3. Discussion

Zinner syndrome is a rare syndrome that is often asymptomatic, and thus, the exact incidence is unknown. Some of the symptoms that have been attributed to Zinner syndrome include urinary urgency, bladder outlet obstruction, and haematospermia [1]. Occasionally, there can be flank or groin pain associated with the cysts also [2]. The cysts can grow to be quite large, and the cyst we present in this case is amongst the largest, reported at 14 cm in maximal diameter. Indeed, this is the first reported case whereby renal transplantation has been precluded by the presence of the cyst.

Zinner syndrome arises due to abnormal development of the Wolffian duct, also known as the mesonephric duct. These usually develop into the epididymis, vas deferens, ejaculatory duct, and seminal vesicle, in response to testosterone around the 8^th^ week of foetal life. Abnormalities in the development of the Wolffian duct can lead to seminal vesicle cyst formation, but there are also implications for renal development. The foetal kidney develops from the metanephric mass of intermediate mesoderm following induction by the metanephric diverticulum, or ureteric bud, which is an outgrowth from the Wolffian duct [3].

Management of these seminal vesicle cysts depends on their presentation and symptoms. There have been approximately 200 cases in the literature with marked variation across the reports. Many of the published case reports are of symptomatic seminal vesicle cysts, and most have had some intervention. There are reports of open, laparoscopic, and robotically assisted excision of the cysts [4, 5], and the patients have all experienced a resolution of their symptoms after treatment. Aspiration of symptomatic cysts, however, tends to lead to recurrence of the symptoms, ultimately requiring surgical excision at a later date [6–8]. In patients where infertility was the presenting complaint, transurethral resection of the ejaculatory ducts was attempted [9], with subsequent improvement in semen characteristics.

Observation is advocated for many asymptomatic patients, as was the case in our patient at the time of his initial diagnosis. However, the method and duration of observation are not specified, and indeed, the natural history of these cysts is unclear. The vast majority of cysts are benign in nature, and malignant change has only been documented in a single case [10]. Most papers that report on safe observation have no follow-up documented beyond 1 year, based on clinical follow-up without surveillance imaging. The longest follow-up reported prior to our report was 11 years for a child with a seminal cyst initially diagnosed as a ureterocele at the age of 3 but reclassified as a seminal vesicle cyst at age 14 [11]. There was no change in size of the cyst noted in that report, and annual clinical surveillance is ongoing. Our paper has the longest follow-up of a case of Zinner syndrome in an adult and is also one of the first to document a significant change in the size of the cyst over time, while remaining asymptomatic; our patient's cyst increased in volume by nearly 80%. There may be a role for radiological surveillance of seminal vesicle cysts given the size changes noted in our paper and the potential malignant risk, but prospective studies would be required to elucidate this fact.

In summary, we present the case of a 58-year-old man with the longest documented follow-up of an enlarging yet asymptomatic seminal vesicle cyst as part of a Zinner syndrome diagnosis, which was excised laparoscopically successfully to facilitate renal transplantation.

## Data Availability

Data are available on request.
